# Epimedii Folium Supplementation Improves Semen Quality, Hormonal Profile, and Immune Function by Modulating Gut Microbiota and Seminal Metabolites in Aged Boars

**DOI:** 10.3390/ani16121833

**Published:** 2026-06-14

**Authors:** Bin Ran, Shengxin Luo, Chenxi Zhou, Long Wen, Junjie Wu, Yunxiang Zhao, Xiaoping Zhu, Zhili Li, Mengjie Liu

**Affiliations:** 1 School of Animal Science and Technology, Foshan University, Foshan 528225, China; 2 College of Animal Science and Technology, Guangxi University, Nanning 530004, China

**Keywords:** Epimedii Folium, reproductive hormones, immune function, multi-omics

## Abstract

This study aimed to investigate the effects of dietary Herba Epimedii Folium (HEF) supplementation on semen quality, reproductive hormones, immune parameters, gut microbiota, and seminal plasma metabolites in aged boars, and to evaluate its potential for extending their reproductive lifespan. A total of 18 Bama boars (approximately 3 years of age) were randomly assigned to three groups (*n* = 6 per group). The control group received a basal diet, while the treatment groups were fed the basal diet supplemented with 3 g/kg or 5 g/kg of HEF for 8 weeks. The results showed that adding HEF to the diet of aged boars increased the motility and concentration of their sperm and reduced the proportion of abnormal sperm. Treatment with 3 g/kg HEF increased serum LH and IgG levels, whereas the 5 g/kg dose elevated IgA levels in both serum and seminal plasma, as well as IgG levels in seminal plasma. Furthermore, 16S rRNA sequencing revealed that dietary HEF supplementation reduced the relative abundance of *Streptococcus* and *Oscillospiraceae UCG-002* in the gut of aged boars. PICRUSt2 analysis predicted that pathways involved in lysine biosynthesis, arginine and proline metabolism, glycine, serine and threonine metabolism, and amino acid-related enzymes were enriched in the HEF treatment group. Semen metabolite profiling showed that the HEF treatment enriched several key metabolites, including 5-hydroxytryptophan, acetylcarnitine, tretinoin, methyltestosterone, prostaglandin A3, and prostaglandin B2. Spearman correlation analysis revealed a negative association between *Streptococcus* abundance and sperm motility, whereas acetylcarnitine, 5-hydroxytryptophan, and prostaglandin A3 were positively associated with motility. Furthermore, 5-hydroxytryptophan levels were positively linked to both sperm concentration and serum LH. In summary, our study demonstrates that Epimedii Folium may enhance the semen quality of aged Bama boars by improving the intestinal microbiota and the metabolic profile of seminal plasma. These findings may offer a theoretical basis for optimizing reproduction and conserving germplasm resources in aged Bama miniature pigs.

## Simple Summary

Boar reproductive performance and the extension of their productive lifespan are critical determinants of production efficiency in the swine industry. Maintaining gut microbiota homeostasis and a favorable seminal plasma metabolic profile is crucial for the reproductive performance of boars. Herba Epimedii Folium (HEF), as a type of functional botanical supplement, is traditionally recognized for its efficacy in treating osteoporosis, delaying aging, and enhancing male sexual function. This study demonstrates that HEF may enhance semen quality and immune status in aged Bama boars. Among them, 3 g/kg HEF increases the serum hormone (LH) level. Integrative multi-omics analysis of intestinal microbiota composition and seminal plasma metabolites in aged boars revealed that HEF promoted intestinal health by reducing the abundance of the potentially pathogenic bacterium *Streptococcus*. Concurrently, HEF modulated the seminal plasma metabolic profile by elevating the levels of 5-hydroxytryptamine, acetylcarnitine, retinoic acid, methyltestosterone, and prostaglandin. This study supports the potential application of HEF in optimizing the reproductive performance of aged boars and conserving germplasm resources.

## 1. Introduction

Semen quality serves as a key proxy for boar fertility due to its strong correlation with fertility outcomes, directly influencing sow reproductive performance and piglet production [1]. However, the productive lifespan of breeding boars is limited, as advancing age is widely recognized to compromise semen quality [2]. The Bama Xiang pig, an indigenous Chinese breed, holds a unique position among native swine due to its compact size and characteristic ‘two-ended black’ coloring [3]. While this breed is renowned for early puberty, superior meat quality, adaptability, and disease resistance, it faces significant reproductive challenges in practical applications. Specifically, Bama boars frequently display reduced libido and an age-related decline in reproductive performance, which significantly limits their production efficiency and utilization in breeding programs [4]. Consequently, extending the reproductive longevity of high-performing boars offers significant economic and genetic benefits by reducing replacement costs, preserving valuable genetics, and maintaining herd fertility.

Ample evidence suggests that herbal supplementation improves semen quality by increasing ejaculate volume and sperm motility while reducing the rate of sperm abnormalities [5]. *Herba epimedii* (*H. epimedii*), commonly known as ‘yin yang huo’ or ‘horny goat weed’, is a traditional Chinese medicine widely used to treat osteoporosis and enhance male sexual function [6]. In China, crude extracts of *Herba epimedii* are also frequently employed as herbal tonics for managing age-related diseases [7]. Notably, icariin, a flavonol glycoside isolated from *Epimedium* species, exhibits testosterone-mimetic properties and has shown potential in improving erectile function in aged and diabetic male rats [8]. Similarly, the total flavones of *Epimedium* demonstrate potent anti-aging effects, significantly promoting longevity in mice [9].

Aging induces significant hormonal and cellular changes in males, which alter sperm quality and fertilization capacity [10]. Seminal plasma serves as the immediate microenvironment for sperm survival and function; metabolites within this fluid, such as amino acids and fatty acids, are essential for sperm energy production, motility, and metabolic activity [11]. Furthermore, emerging evidence highlights that the gut–testis axis may be a potential mediator of herbal efficacy. This biological pathway serves as a complex communication network where gut microbiota may also influence sperm quality by actively modulating key physiological processes, specifically including energy metabolism, spermatogenesis, and hormone production [12]. However, research focusing on prolonging the reproductive lifespan of breeding boars while simultaneously enhancing semen quality remains limited. Toward this end, this study investigated the effects of HEF on semen quality, reproductive hormones, immune parameters, intestinal microbiota, and seminal plasma metabolites in aged Bama miniature pigs using a multi-omics approach. These findings provide a theoretical basis for optimizing the reproductive performance of aged boars and conserving germplasm resources.

## 2. Materials and Methods

### 2.1. Preparation and Qualitative Analysis of Herba Epimedii Folium (HEF)

Herba Epimedii Folium (HEF) was procured from Guangdong Benyuan Technology Development Co., Ltd. The preparation of the extract was carried out according to the method previously reported by Wu et al. [13]. In brief, Epimedii Folium was extracted via double decoction in distilled water (0.1 g/mL, 1.5 h per cycle). The combined extracts were concentrated by rotary evaporation, lyophilized to a solid, ground into powder, and stored at −20 °C. According to the method previously reported by Chen et al. [14], the chemical and active component profiles of the HEF extract were identified using liquid chromatography–mass spectrometry (LC-MS).

### 2.2. Animals and Experimental Design

The Bama miniature boars used in this study were obtained from Bama Original Breed Pig Farming and Industrial Co., Ltd. (Nanning, China). A total of 18 Bama boars, approximately 3 years of age with an average body weight of 40.5 ± 1.3 kg, were selected for the experiment and randomly assigned to three groups (*n* = 6 per group). There were no significant differences in the semen quality of boars among the groups before randomization. The control (CON) group received a basal diet, while the treatment groups were fed the basal diet supplemented with 3 g/kg (EFL) or 5 g/kg (EFH) of HEF for 8 weeks. The Bama miniature boars were individually housed in pens measuring 2.2 m × 0.6 m, fed twice daily at 07:00 and 14:00 h, and provided with ad libitum access to water. Boars were kept under controlled environmental conditions (20 ± 3 °C, 60–75% humidity). Diet composition and chemical composition are shown in Supplementary Table S1.

### 2.3. Sample Collection and Semen Quality Assessment

During the sampling period, semen, blood, and fecal samples were collected from each boar. Semen was harvested using the dummy mount technique with an artificial vagina. The gel fraction was immediately removed by filtration through sterile gauze, and the gel-free volume was recorded. Semen quality parameters, including volume, sperm concentration, motility, and abnormality rates, were assessed following established protocols [15]. Subsequently, ejaculates were centrifuged at 3000× *g* for 10 min to separate the seminal plasma, which was aliquoted and stored at −80 °C. Blood samples were collected via venipuncture of the hindlimb vein into sterile collection tubes. After clotting at room temperature, samples were centrifuged at 3000× *g* for 10 min to obtain serum. The serum samples were stored at −80 °C. Fresh fecal samples were obtained via digital rectal stimulation, immediately placed in sterile tubes on ice, and stored at −80 °C until analysis.

### 2.4. Detection of Reproductive Hormones and Immune-Related Indicators

Serum concentrations of luteinizing hormone (LH), follicle-stimulating hormone (FSH), and testosterone were determined using commercial pig-specific ELISA kits purchased from Shanghai Enzyme-Linked Biotechnology Co., Ltd. (Shanghai, China), following the manufacturer’s instructions. Serum biochemical parameters such as total protein (TP) and albumin (ALB) were analyzed using a biochemical analyzer (BS2000M2, Shenzhen, China). Serum and seminal immunoglobulin (IgA, IgG, and IgM) concentrations and seminal TP and ALB contents were determined using commercial pig ELISA kits (Jiangsu Jingmei Biotechnology Co., Ltd.; Yancheng, China).

### 2.5. Boar Feces 16s RNA Sequencing

Total genomic DNA was isolated from fecal samples (*n* = 4) using the QIAamp DNA Stool Mini Kit (QIAGEN, Hilden, Germany) per the manufacturer’s guidelines. PCR amplification of the 16S rRNA gene V3–V4 region was performed using the universal bacterial primers 338F (5′-ACTCCTACGGGAGGCAGCAG-3′) and 806R (5′-GGACTACHVGGGTWTCTAAT-3′). PCR amplification was conducted as previously described [16]. Amplicons were resolved on a 2% agarose gel, and the target fragments were purified using the AxyPrep DNA Gel Extraction Kit (Axygen, Union City, CA, USA). Subsequently, high-quality sequences were clustered into operational taxonomic units (OTUs) at a 97% similarity threshold. The sequencing was carried out by Novogene Bioinformatics Co., Ltd. (Tianjin, China) using the Illumina NovaSeq 2000 platform, and microbial analysis was performed on the NovoMagic Cloud platform (https://magic-plus.novogene.com/).

### 2.6. Seminal Plasma Metabolome Assay by LC-MS/MS

Seminal plasma samples were retrieved from storage at −80 °C and thawed on ice. The samples were randomly selected from each group (*n* = 4). The proteins were precipitated by adding four volumes of cold methanol containing 0.1% formic acid to the samples. After vortexing, the mixtures were incubated at −20 °C for 30 min and then centrifuged at 12,000× *g* for 10 min at 4 °C. The resulting supernatant was collected and analysed using an LC-MS system. Chromatographic separation was performed on an ACQUITY UPLC BEH C18 column (1.7 µm, 2.1 mm × 100 mm; Waters, Milford, MA, USA), which was maintained at 45 °C. The mobile phase consisted of (A) water containing 0.1% formic acid and (B) acetonitrile containing 0.1% formic acid. The gradient elution programme was as follows: 0–2 min: 5–20% B; 2–4 min: 20–60% B; 4–11 min: 60–100% B; 11–13 min: held at 100% B; 13–13.5 min: 100–5% B; 13.5–14.5 min: held at 5% B. The flow rate was set at 0.4 mL/min and the injection volume at 5 µL. Samples were maintained at 4 °C in the autosampler. Liquid Chromatography–Tandem Mass Spectrometry (LC-MS/MS) data were acquired using electrospray ionisation (ESI) in both positive and negative ion modes.

### 2.7. Statistical Analysis

Data were organized using Microsoft Excel and statistically analyzed using SPSS version 20.0 (IBM Corp., Armonk, NY, USA) and GraphPad Prism version 9.0 (GraphPad Software, San Diego, CA, USA). Differences among multiple groups were evaluated by one-way analysis of variance (ANOVA), followed by Duncan’s new multiple range test for post hoc comparisons. A *p*-value < 0.05 was considered to indicate a statistically significant difference.

## 3. Results

### 3.1. The Chemical Components of HEF

**Figure 1:**
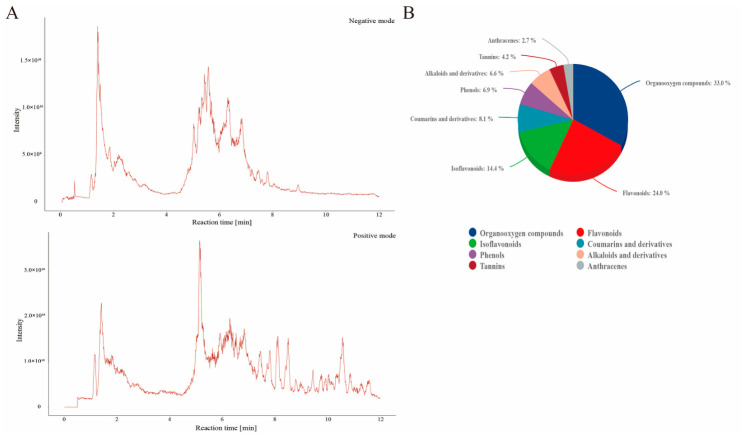
LC-MS analysis of HEF. (**A**) Base peak ion (BPI) chromatograms of HEF obtained in negative and positive ion modes. (**B**) Distribution of the identified HEF metabolites by chemical class.

As shown in Figure 1A, the base peak ion (BPI) chromatograms of HEF were acquired in both negative and positive ion modes via LC-MS. Representative chemical markers identified in HEF included Icariside I, Epimedoside A, Sagittatoside B, Baohuoside I, Icariin, Epimedin A, Epmedin C, Epimedin B, and Noricaritin (Supplementary Table S2). A total of 591 compounds were identified, with the predominant bioactive classes being flavonoids, isoflavonoids, coumarins, phenols, alkaloids, and tannins (Figure 1B).

### 3.2. Effect of Dietary HEF on the Semen Quality of Aged Bama Boars

The effect of HEF treatment on the semen quality of aged Bama boars is shown in Table 1. Compared with the CON group, the EFL and EFH groups significantly reduced the abnormal sperm rate but increased sperm motility and sperm concentration (*p* < 0.05). Among them, the EFL group had the most obvious effect on improving sperm motility, while the EFH group had the most significant effect on reducing the abnormal sperm rate. There was no significant difference in the sperm volume of the boars among the groups (*p* > 0.05).

### 3.3. Effect of Dietary HEF on the Reproductive Hormones of Aged Bama Boars

The effect of HEF treatment on the serum reproductive hormone levels of aged Bama boars is shown in Table 2. Compared with the CON group, the EFL group significantly increased the serum LH level (*p* < 0.05). However, there were no significant differences in the serum FSH and testosterone levels among all groups (*p* > 0.05).

### 3.4. Effect of Dietary HEF on the Immune-Related Indicators of Aged Bama Boars

The effects of HEF treatment on the immune indicators of serum and seminal plasma in aged Bama boars are shown in Table 3. In the serum of boars, compared with the CON group, the EFL group significantly increased the level of serum IgG (*p* < 0.05) and showed an upward trend in the level of serum TP (*p* = 0.073), while the EFH group significantly increased the level of IgA (*p* < 0.05). The level of serum ALB in the EFL group was significantly higher than that in the CON group and the EFH group (*p* < 0.05). The EFH group had significantly higher levels of IgA, IgG, and TP in the seminal plasma compared to the CON group. However, there were no significant differences among the groups in the levels of serum IgM and TP, or in the levels of seminal plasma IgM and ALB (*p* > 0.05).

### 3.5. Effects of Dietary HEF on the Diversity and Composition Structure of the Intestinal Flora in Aged Bama Boars

**Figure 2:**
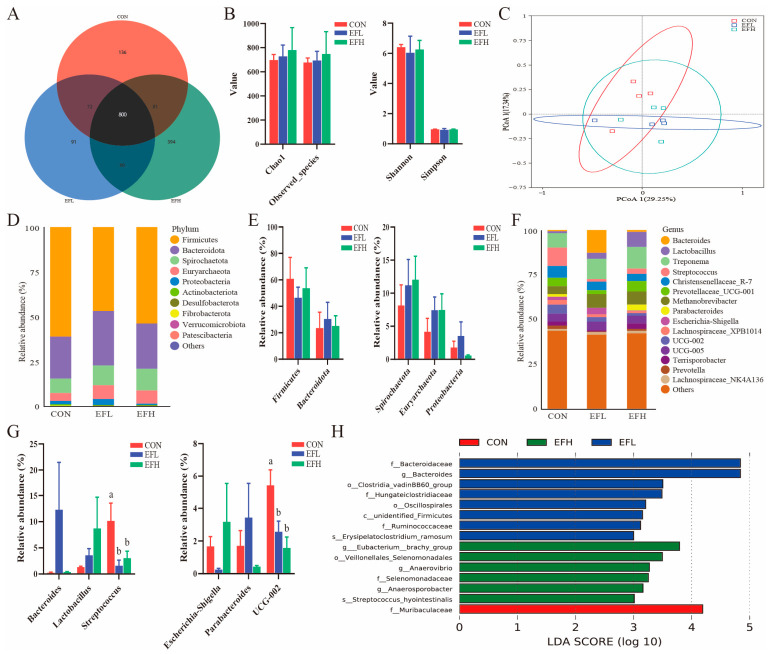
Effects of dietary HEF supplementation on the composition and diversity in the fecal microbiota of aged Bama boars. (**A**)Venn diagram illustrating shared and unique OTUs. (**B**) Bacterial alpha-diversity indices (Chao1, Observed_species, Shannon, and Simpson). (**C**) Principal coordinate analysis (PCoA) scatterplot. (**D**,**F**) Relative abundance of fecal microbiota at the phylum and genus level. (**E**,**G**) Comparison of dominant fecal microbiota at the phylum and genus. (**H**) Linear discriminant analysis Effect Size (LEfSe) bar based on phylum to genus level (LDA > 3). EFL: low-dose HEF (3 g/kg); EFH: high-dose HEF (5 g/kg). Different lowercase letters indicate statistically significant differences (*p* < 0.05, *n* = 4).

The effect of HEF treatment on the intestinal microbiota of Bama boars is shown in Figure 2. The Venn diagram analysis (Figure 2A) shows that there are 800 shared OTUs in the three groups, while the unique OTUs in the CON, EFL, and EFH groups are 136, 91, and 394, respectively. The α-diversity indices (Shannon, Simpson, Chao1, Observed_species) showed no significant differences among the groups (Figure 2B). PCoA analysis based on the Bray–Curtis distance indicates that there is partial overlap between the control group and the EFL group, as well as the EFH group, while the EFL group and the EFH group are closely clustered together (Figure 2C). The dominant microbiota in all groups mainly included *Firmicutes*, *Bacteroides*, *Spirochaetota*, *Euryarchaeota*, and *Proteobacteria* at the phylum level, but there were no significant differences among the groups (Figure 2D,E). The analysis at the genus level showed that *Bacteroides*, *Lactobacillus*, *Treponema*, and *Streptococcus* were the main genera (Figure 2F). The difference analysis further revealed that the abundance of *Streptococcus* and *Oscillospiraceae UCG-002* in the EFL and EFH groups was significantly lower than that in the CON group (Figure 2G, *p* < 0.05). LEfSe analysis identified 16 biomarkers with intergroup differences (LDA > 3.0, *p* < 0.05), among which the EFH group was enriched with *f_Bacteroidaceae* and *g_Bacteroides* (Figure 2H). The EFL group was enriched with *g_Eubacterium_brachy_group* and *o_Veillonellales-Selenomonadales*, while the CON group was enriched with *f_Muribaculaceae*.

### 3.6. Functional Prediction of Gut Microbiota

**Figure 3:**
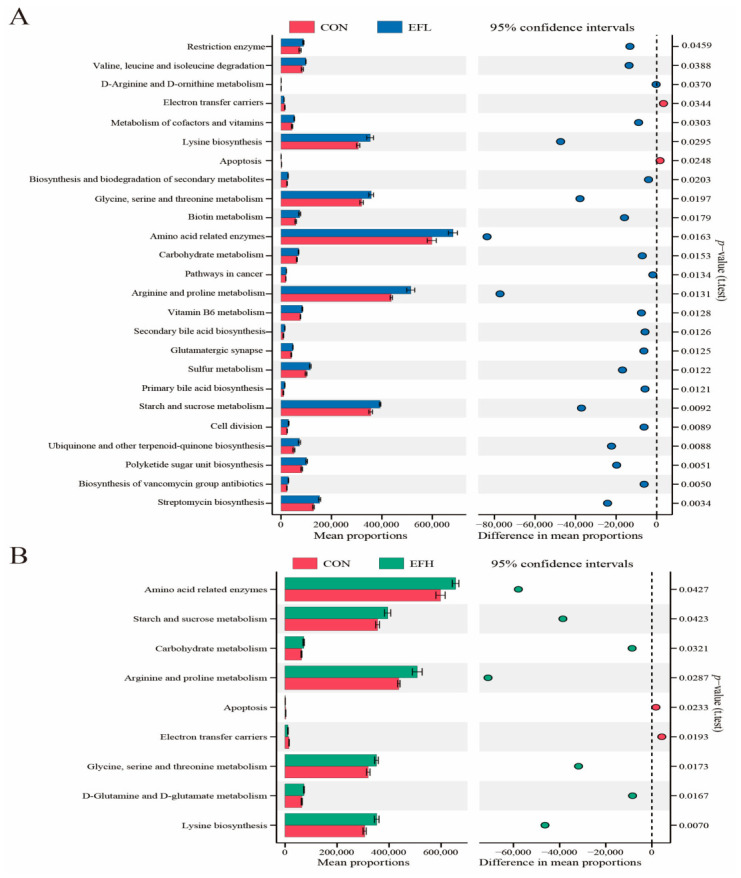
KEGG pathway enrichment (level 3) based on Phylogenetic Investigation of Communities by Reconstruction of Unobserved States (PICRUSt2) prediction for three groups. (**A**,**B**) The average abundance of the differences in functional pathways among CON vs. EFL and CON vs. EFH. EFL: low-dose HEF (3 g/kg); EFH: high-dose HEF (5 g/kg). (*p* < 0.05, *n* = 4).

Based on the 16S rRNA gene sequence, PICRUSt2 was used to predict the intestinal flora function, and the functional annotation was completed through the KEGG database (Level 3). The average abundance of the main functional pathways among the three groups is shown in Figure 3. A total of 25 enriched functional pathways were identified in the CON group and the EFL group, mainly concentrated in amino acid metabolism, cell growth and death, carbohydrate metabolism, metabolism of other amino acids, lipid metabolism, energy metabolism, metabolism of cofactors and vitamins, and metabolism of terpenoids and polyketides (Figure 3A). Compared with the CON group, the EFL group significantly downregulated the apoptosis pathway but upregulated the amino acid-related enzymes, arginine and proline metabolism, biotin metabolism, D-arginine and D-ornithine metabolism, glycine, serine and threonine metabolism, lysine biosynthesis, primary bile acid biosynthesis, sulfur metabolism, valine, leucine and isoleucine degradation, and vitamin B6 metabolism pathways. A total of 9 enriched functional pathways were identified in the CON group and the EFH group, mainly concentrated in amino acid metabolism, carbohydrate metabolism, metabolism of other amino acids, and cell growth and death (Figure 3B). Difference analysis indicated that the EFH group significantly upregulated the pathways of amino acid-related enzymes, arginine and proline metabolism, glycine, serine and threonine metabolism, lysine biosynthesis, and starch and sucrose metabolism, while downregulating the apoptosis pathway.

### 3.7. HEF Improved the Seminal Plasma Metabolites of Aging Boars

**Figure 4:**
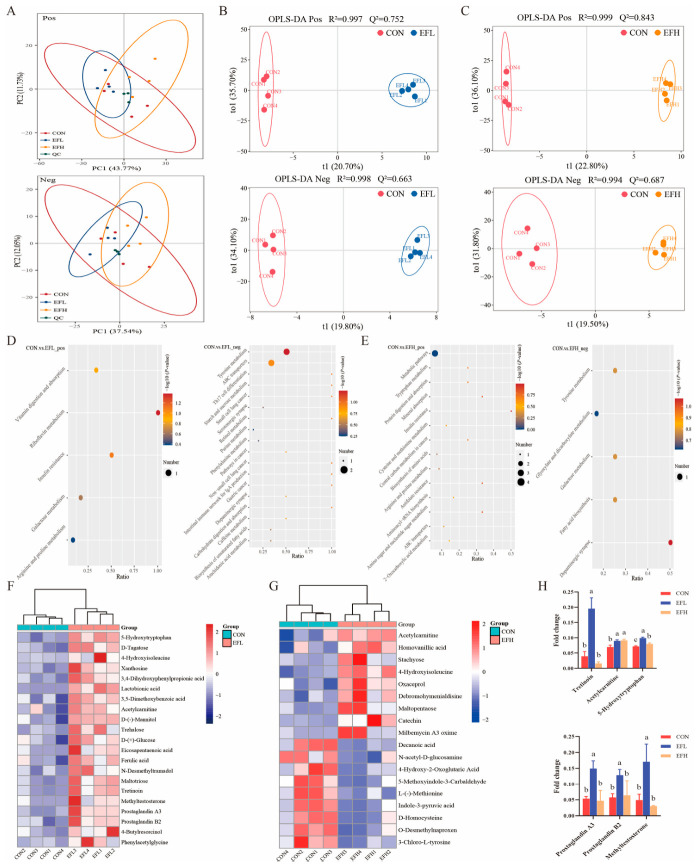
HEF improved the seminal plasma metabolites of aging boars. (**A**) Plot of PCA scores for three groups in cation and anion mode. (**B**) Plot of OPLS-DA scores under cations and anions in CON vs. EFL. (**C**) Plot of OPLS-DA scores under cations and anions in CON vs. EFH. (**D**,**E**) KEGG enrichment analyses of the differentially enriched pathways under cations and anions (CON vs. EFL and CON vs. EFH). (**F**,**G**) Cluster heat maps of differentiated metabolites regulated by HEF (CON vs. EFL and CON vs. EFH). (**H**) The most representative metabolites that show the most significant differences among the three groups. EFL: low-dose HEF (3 g/kg); EFH: high-dose HEF (5 g/kg). The different superscript small letters were judged as a significant difference (*p* < 0.05, *n* = 4).

Non-targeted metabolomics technology was employed to analyze the seminal plasma samples of male pigs from different groups, in order to investigate the effects of HEF on sperm metabolites (Figure 4). The principal component analysis results in both cationic and anionic modes showed that there was a certain degree of separation among the samples of the CON group, the EFL group, and the EFH group, and the aggregation of the quality control samples was good, indicating that the experiment had good repeatability and stability. OPLS-DA analysis showed that in both ion patterns, the CON vs. EFL and CON vs. EFH comparison groups were significantly separated, indicating that HEF has a positive effect on sperm metabolites. The OPLS-DA models were authenticated by cross-validation among CON vs. EFL (R^2^  =  0.997 and Q^2^  =  0.752 for positive ion modes and R^2^  =  0.998 and Q^2^  =  0.663 for negative ion modes) and CON vs. EFH (R^2^  =  0.999 and Q^2^  =  0.843 for positive ion modes and R^2^  =  0.994 and Q^2^  =  0.687 for negative ion modes). The value of R^2^Y was close to 1 for these cationic and anionic modes, and Q^2^ was greater than 0.5, suggesting that this model was reliable and effective. In the cation and anion modes of the CON group and the EFL group (Figure 4D), these differential metabolites were mainly concentrated in the KEGG pathways related to vitamin digestion and absorption, riboflavin metabolism, tyrosine metabolism, and ABC transporters. As shown in Figure 4F, the heatmap of the changes in differential metabolites indicates that the EFL group mainly upregulated levels of 21 metabolites, including 5-hydroxytryptophan, lactobionic acid, acetylcarnitine, tretinoin, methyltestosterone, prostaglandin A3, prostaglandin B2, and phenylacetylglycine, and so on. In the cation and anion modes of the CON group and the EFL group (Figure 4E), these differential metabolites were mainly concentrated in the KEGG pathways related to tryptophan metabolism, protein digestion and absorption, tyrosine metabolism, and galactose metabolism. As shown in Figure 4G, the heatmap of the changes in differential metabolites indicates that the EFH group mainly increased the levels of 9 metabolites (acetylcarnitine, homovanillic acid, and 4-hydroxyisoleucine) and mainly decreased the levels of 9 metabolites, including *N*-acetyl-D-glucosamine, L-(-)-methionine, and indole-3-pyruvic acid. As shown in Figure 4H, six metabolites exhibited differential expression among the three groups: 5-hydroxytryptophan, acetylcarnitine, tretinoin, methyltestosterone, prostaglandin A3, and prostaglandin B2. The levels of acetylcarnitine and 5-hydroxytryptophan in the EFL and EFH groups were significantly higher than those in the CON group (*p* < 0.05). The levels of tretinoin, prostaglandin A3, prostaglandin B2, and methyltestosterone in the EFL group were significantly higher than those in the CON group (*p* < 0.05).

### 3.8. Spearman Correlation Among Fecal Microbes, Seminal Plasma Metabolites, and Different Parameters

**Figure 5:**
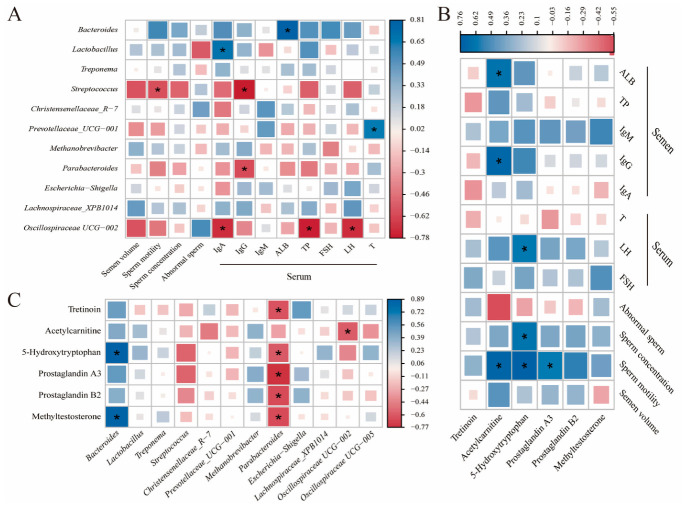
Correlations among fecal microbes, seminal plasma metabolites, sperm parameters, and serum hormones. (**A**) The correlation between gut microbiota and semen quality, as well as serum parameters; (**B**) The correlations between intestinal fecal metabolites and semen quality, semen parameters, and serum hormones; (**C**) The correlation between intestinal fecal metabolites and intestinal microbiota. Blue squares represent positive correlation, and red squares represent negative correlation. The shade of color of the squares represents the strength of the correlation (the darker the color of the square, the stronger the correlation). * indicates *p* < 0.05.

The Spearman correlation analysis (Figure 5) indicated that the fecal microbiota, sperm metabolites, semen parameters, and serum parameters were well correlated. First, there was a good correlation among the gut microbes, semen quality, and serum parameters. The relative abundances of *Bacteroides* and *Lactobacillus* were positively correlated with the levels of IgA and ALB in the serum, respectively. The relative abundance of *Streptococcus* and *Parabacteroides* was negatively correlated with the level of IgG in the serum, and the relative abundance of *Streptococcus* was also negatively correlated with sperm motility. The relative abundance of *Oscillospiraceae UCG-002* was negatively correlated with the levels of IgA, TP, and LH in the serum, while *Prevotellaceae_UCG-001* was negatively correlated with the level of T (Testosterone) in the serum. Second, there was a good correlation between sperm metabolites and gut microbes. The relative abundance of acetylcarnitine is negatively correlated with that of *Oscillospiraceae UCG-002*. The relative abundance of *Parabacteroides* is negatively correlated with the levels of tretinoin, 5-hydroxytryptophan, prostaglandin A3, prostaglandin B2, and methyltestosterone, while the relative abundance of *Bacteroides* is positively correlated with the levels of 5-hydroxytryptophan and methyltestosterone. Furthermore, there were good correlations among the sperm metabolites, semen quality, semen parameters, and serum reproductive hormones. The levels of acetylcarnitine, 5-hydroxytryptophan, and prostaglandin A3 were positively correlated with sperm motility. Among them, 5-hydroxytryptophan was also positively correlated with sperm concentration and the level of serum LH. Meanwhile, the level of acetylcarnitine was positively correlated with the levels of IgG and ALB in semen.

## 4. Discussion

*Herba epimedii*, a cornerstone of Traditional Chinese Medicine (TCM), is valued for its anti-inflammatory, antioxidant, and hormone-regulating properties that combat aging and enhance vitality [17]. In this experiment, adding Epimedii Folium to the diet of aged boars resulted in improved sperm motility and sperm concentration, and a reduction in the ratio of abnormal sperm. Research indicates that total flavonoids of Epimedium protect the male reproductive system from structural and functional damage, thereby boosting sperm quantity and quality [18]. Icariin, especially, effectively ameliorated aging-associated testicular dysfunction in aged mice by restoring testicular weight and index, enhancing sperm concentration and viability, and increasing spermatogenic cell populations [19]. Previous studies have confirmed these anti-aging benefits: a 4-month dietary supplementation with icariin from *Epimedium* significantly attenuated age-related declines in testicular function, evidenced by increased testicular and epididymal weights/indices, improved sperm parameters, and enlarged seminiferous tubule diameters and epithelial heights [20]. From this, it can be inferred that the HEF treatment may have improved the semen quality of the aged Bama miniature boars.

Previous studies have established that fluctuations in reproductive hormone levels significantly compromise reproductive capacity in animals. In this context, *Epimedium* has been shown to restore these hormone levels, thereby effectively enhancing sexual function [21]. In our current experiment, adding Epimedii Folium to the diet of aged boars increased the serum LH level. Studies have shown that administering icariin at 50 mg/kg body weight for 80 days improved the reproductive performance of male dairy goats. This improvement was characterized by elevated serum levels of gonadotropin-releasing hormone (GnRH), luteinizing hormone (LH), and testosterone, as well as enhanced spermatogenesis and sperm motility [22]. Similarly, polyphenolic compounds in Epimedium were found to significantly increase reproductive hormone levels, such as LH, in albino rats [23]. Beyond its reproductive benefits, *Epimedium* also modulates immune function. Serum immunoglobulins (IgA, IgG, and IgM) serve as vital biomarkers of immune status and play essential roles in defense against infections [24]. In this experiment, the addition of Epimedii Folium to the diet of aged boars increased the levels of immunoglobulins (IgA and IgG). Similar to these findings, previous studies reported that sulfated *Epimedium* polysaccharides elevated concentrations of IL-10, total IgG, and IgA [25]. Collectively, these results suggest that HEF may enhance both reproductive hormone levels (specifically LH) and immune status in aged Bama miniature boars, thereby contributing to improved semen quality.

A previous study reported that age-related changes in semen quality are associated with changes in the composition of seminal plasma [26]. We conducted a non-targeted metabolomics analysis on the seminal plasma components of Bama boars treated with HEF to identify the key metabolic pathways. In this current experiment, the addition of HEF improved the semen metabolic profile of aged boars, mainly increasing the levels of metabolites such as 5-hydroxytryptophan, acetylcarnitine, tretinoin, methyltestosterone, prostaglandin A3, and prostaglandin B2. Methyltestosterone (MT), a potent synthetic androgen derived from testosterone, is primarily prescribed for the treatment of hypogonadism and certain growth disorders [27]. Similarly, recent studies have shown that supplementing tretinoin-loaded solid lipid-core nanocapsules (TTN-SLN) enhances sperm viability and motility parameters (total and progressive), and concomitantly reduces DNA fragmentation [28]. 5-Hydroxytryptamine (5-HT, also known as serotonin) is indeed present in sperm and directly participates in regulating key sperm physiological functions—particularly sperm hyperactivation [29]. In the current experiment, acetylcarnitine, 5-hydroxytryptophan, and prostaglandin A3 were positively correlated with sperm motility. Notably, 5-hydroxytryptophan also exhibited significant positive correlations with sperm concentration and serum luteinizing hormone (LH) levels. L-carnitine facilitates the β-oxidation of long-chain fatty acids, and as its active metabolite, L-acetylcarnitine, serves as a vital antioxidant protecting sperm mitochondria from oxidative stress [30]. Apart from their recognized roles in inflammation (particularly for prostaglandins), both prostaglandins and polyamines are key bioactive molecules in semen, significantly influencing sperm quality [31]. Collectively, these findings suggest that the potential mechanism by which HEF may extend the reproductive lifespan of aging Bama miniature boars involves restoring seminal metabolic homeostasis, thereby enhancing overall semen quality.

Gut microbiota diversity is closely linked to host health, and *Epimedium* has been shown to improve animal health by modulating microbial composition [32]. In this study, *Firmicutes*, *Bacteroidota*, and *Spirochaetota* were the most abundant phyla in the intestines of aged boars, with *Firmicutes* being the dominant phylum, consistent with previous reports [33]. Furthermore, adding Epimedii Folium to the diet reduced the relative abundance of *Proteobacteria* in the intestines of aged boars. This is similar to the findings of the research conducted by Xie et al. [32], where *Epimedium* enhanced microbial richness and diversity by increasing beneficial *Firmicutes* while suppressing potentially pathogenic *Proteobacteria*. Meanwhile, we observed that feeding HEF to the aged boars’ diet reduced the relative abundance of *Streptococcus* and *Oscillospiraceae UCG-002*. The correlation analysis revealed that the abundance of *Streptococcus* was negatively correlated with sperm motility. *Streptococcus* (phylum *Bacillota*, formerly *Firmicutes*) is widely distributed in nature and hosts and is implicated in various inflammatory conditions [34]. Both *Streptococcus* and *Klebsiella* have been associated with impaired sperm function, reducing motility and inducing apoptosis [35]. Feeding aged microminipigs a polyphenol-rich diet supplemented with sweet potato powder led to a significant reduction in the relative abundance of *Oscillospiraceae UCG-002* [36]. ICA (icariin), a compound found in plants, can improve the health of the liver, kidney, and intestines in aged mice by altering the microbiota composition to resemble that of younger mice [37]. These results suggest that HEF may improve reproductive health in aged boars partly by optimizing gut microbiota structure. This observation may primarily be attributed to the pivotal regulatory role of gut microbiota at the diet–host interface, which facilitates inter-organ communication, particularly along the gut–testis axis. However, the relationship and mechanism by which HEF regulates the changes in the intestinal flora of boars and its impact on semen quality still require further investigation.

Gut microbiota and their metabolites play a pivotal role in promoting glycolysis to maintain sperm energy supply and enhance motility. For instance, leucine supplementation has been shown to improve sperm curvilinear velocity in boars [38]. In this study, PICRUSt2 functional prediction revealed that dietary supplementation with Epimedii Folium in aged boars significantly upregulated intestinal microbial metabolic pathways. These enriched pathways were primarily associated with amino acid metabolism, including arginine and proline metabolism, glycine, serine, and threonine metabolism, and lysine biosynthesis. Similar to our previous findings, differentially abundant taxa in breeding boars were predominantly involved in these beneficial metabolic processes [39]. The significance of these pathways is underscored by evidence that amino acid metabolic disorders are linked to structural and functional defects in spermatozoa, particularly in men with severe oligospermia [40]. Conversely, dietary amino acid supplementation improves sperm quality, modulates seminal plasma composition, and enhances fertility in boars [1]. Furthermore, Epimedium and its bioactive flavonoids are known to ameliorate health status by regulating lipid, energy, and amino acid metabolism [41]. Specifically, these compounds modulate gut microbiota composition and function to improve digestive absorption and immune status, thereby reducing harmful metabolites while increasing the abundance of beneficial metabolites [41,42]. Collectively, these data suggest that HEF may enhance sperm motility in aged boars by restoring gut microbiota-mediated amino acid metabolism. However, the metabolites (such as amino acid metabolism) derived from the intestinal microbiota of the boars by HEF need to be further verified, and the relationship between these metabolites and the quality of semen also requires more in-depth study.

Although this study utilized multi-omics approaches to evaluate the comprehensive effects of HEF on aged Bama miniature boars, future research employing factorial designs is needed to elucidate the specific mechanisms by which key metabolites regulate sperm motility. In the multi-omics analyses, the sample size was limited (*n* = 4), which may reduce the statistical power and limit the generalizability of the findings regarding fecal microbiota and seminal plasma metabolites. These limitations of our study are acknowledged; thus, increasing the sample size of the experiment and investigating the metabolites that affect the quality of semen, such as the content of 5-hydroxytryptophan, acetylcarnitine, methyltestosterone, prostaglandin, etc., are essential in future studies. Furthermore, large-scale production trials involving direct reproductive assessments, such as artificial insemination, are warranted to confirm whether HEF supplementation translates into improved reproductive performance under commercial conditions.

## 5. Conclusions

In conclusion, dietary HEF supplementation improves the semen quality of aged breeding boars by modulating gut microbiota and seminal plasma metabolites in aged Bama miniature boars. Therefore, Epimedii Folium shows great potential as a feed additive to optimize the reproductive performance of Bama miniature pigs and promote the preservation of their germplasm resources.

## Figures and Tables

**Table 1:** Effect of HEF on the semen quality in aged Bama boars.

Items	CON	EFL	EFH	*p*-Value
Sperm volume, mL	158.50 ± 52.42	222.00 ± 43.06	234.75 ± 51.81	0.117
Sperm motility, %	78.79 ± 9.54 ^b^	98.48 ± 0.98 ^a^	97.75 ± 1.30 ^a^	0.001
Sperm concentration, 10^8^/mL	2.51 ± 2.16 ^b^	7.02 ± 1.34 ^a^	6.69 ± 2.79 ^a^	0.030
Abnormal sperm rate, %	4.78 ± 2.28 ^a^	1.22 ± 0.87 ^b^	0.47 ± 0.32 ^b^	0.003

Values with different superscript letters differ significantly (*p* < 0.05; *n* = 6). CON: control group; EFL: low-dose HEF (3 g/kg); EFH: high-dose HEF (5 g/kg).

**Table 2:** Effect of HEF on the serum hormone indicators in aged Bama boars.

Items	CON	EFL	EFH	*p*-Value
FSH (mIU/mL)	8.89 ± 1.12	9.90 ± 0.80	9.11 ± 0.62	0.278
LH (mIU/mL)	10.98 ± 0.31 ^b^	13.90 ± 2.08 ^a^	12.51 ± 1.02 ^ab^	0.040
T (pg/mL)	9.12 ± 0.28	9.56 ± 2.71	9.85 ± 0.77	0.819

Values with different superscript letters differ significantly (*p*< 0.05; *n* = 6). CON: control group; EFL: low-dose HEF (3 g/kg); EFH: high-dose HEF (5 g/kg).

**Table 3:** Effect of HEF on the immune indicators in aged Bama boars.

Items	CON	EFL	EFH	*p*-Value
Serum				
IgA (μg/mL)	7.37 ± 1.47 ^b^	9.15 ± 0.78 ^ab^	10.01 ± 1.45 ^a^	0.045
IgG (g/L)	16.50 ± 1.95 ^b^	24.85 ± 6.16 ^a^	18.70 ± 2.69 ^ab^	0.042
IgM (g/L)	26.41 ± 3.93	27.37 ± 2.08	25.20 ± 2.97	0.623
TP (g/L)	75.01 ± 2.66	83.50 ± 4.81	80.24 ± 6.00	0.083
ALB (g/L)	54.38 ± 1.68 ^b^	61.60 ± 4.65 ^a^	55.28 ± 3.80 ^b^	0.038
Seminal plasma				
IgA (μg/mL)	38.78 ± 6.96 ^b^	47.61 ± 5.82 ^ab^	54.47 ± 6.38 ^a^	0.022
IgG (g/L)	13.88 ± 5.12 ^b^	26.08 ± 3.03 ^ab^	37.05 ± 16.57 ^a^	0.032
IgM (g/L)	57.18 ± 3.38	69.54 ± 14.93	61.43 ± 8.63	0.267
TP (g/L)	1605.33 ± 93.09 ^b^	1724.60 ± 139.06 ^ab^	1957.27 ± 223.88 ^a^	0.036
ALB (g/L)	0.12 ± 0.05	0.21 ± 0.09	0.34 ± 0.23	0.155

Values with different superscript letters differ significantly (*p* < 0.05; *n* = 6). CON: control group; EFL: low-dose HEF (3 g/kg); EFH: high-dose HEF (5 g/kg).

## Data Availability

The names of the repository/repositories and accession number can be found below: https://www.ncbi.nlm.nih.gov/sra/PRJNA1433473.

## Supplementary Materials

The following supporting information can be downloaded at: https://www.mdpi.com/article/10.3390/ani16121833/s1, Table S1: The ingredient and nutrient composition of the basal diet (% as fed basis); Table S2: Representative chemical components of HEF identified by LC-MS.
