# Supplementary material for: Epimedii Folium Supplementation Improves Semen Quality, Hormonal Profile, and Immune Function by Modulating Gut Microbiota and Seminal Metabolites in Aged Boars
Source: Animals (Basel). 2026 Jun 14;16(12):1833. doi: 10.3390/ani16121833 (PMC13295793; doi:10.3390/ani16121833)
Supplement: Supplementary file 1 [file animals-16-01833-s001.zip › animals-4311045-supplementary.pdf]

Supplementary information

Supplementary **Table S1.** The ingredient and nutrient composition of the basal diet (% as fed basis).

Ingredients	Content (%)
Corn	52.88
Soybean meal	13.39
Rice bran	8.57
Whey	8.22
Soybean oil	3.0
Sugar	8
Limestone	1.4
Attapulgite	0.96
Salt	2.20
L-Lysine	0.20
L-Threonine	0.71
Premixes 1	0.47
Total	100
Nutrient composition 2	
Crude protein	13.00
Calcium	0.79
Phosphorus	0.60
Lysine	1.00
Threonine	1.20
Isoleucine	0.49
Methionine & Cysteine	0.43

[1] The premix per kilogram of feed contains vitamin A 4000 IU, vitamin D3 400 IU, vitamin E 18mg, vitamin K3 0.5 mg, vitamin B2 1.5 mg, vitamin B12 8ug, pantothenic acid 1.8 mg, niacin 1.8 mg, Fe 200 mg, Cu 20 mg, Mn 20 mg, Zn 20 mg, I 1mg/kg, Se 0.2mg.

[2] The nutrient levels were calculated from data provided by Feed Database in China.

Supplementary Table S2. Representative chemical components of HEF identified by LC-MS.

Name	Formula	Exact mass	Area	CAS	Structure
Epimedoside A	C32H38O15	661.21527	2226642694.87376	39012-04-9	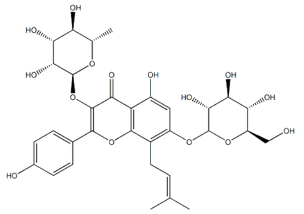
Epimedoside	C37H44O17	759.25283	403166970.769718	106441-31-0	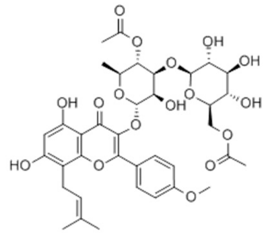
Maohuoside A	C27H32O12	547.18295	60606022.35315	128988-55-6	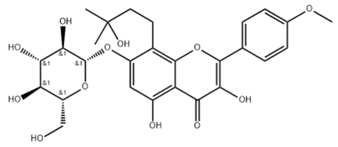
Baohuoside I	C27H30O10	513.17728	1242879965.61097	113558-15-9	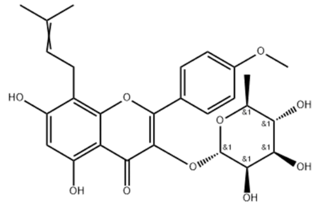
Sagittatoside B	C32H38O14	645.22037	1437873745.88155	118525-36-3	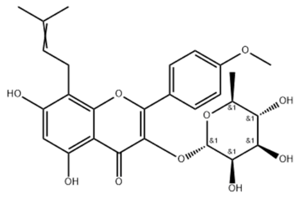
Chrysoeriol	C16H12O6	299.05636	204749084.222468	491-71-4	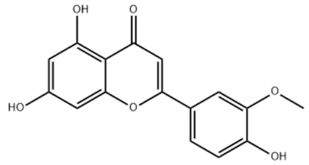
Astragalin	C21H20O11	447.09372	2312620138.94151	480-10-4	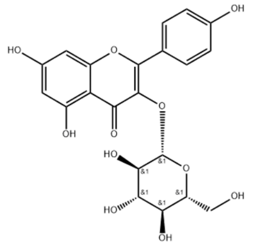
Emodin	C15H10O5	269.04585	595665749.342334	518-82-1	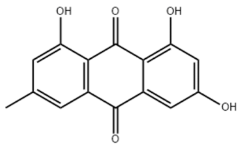

Luteolin	C15H10O6	285.04073	631031762.065228	491-70-3	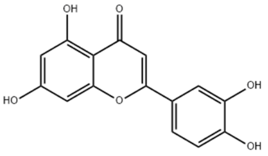
Hyperoside	C21H20O12	463.08882	3640485843.72646	482-36-0	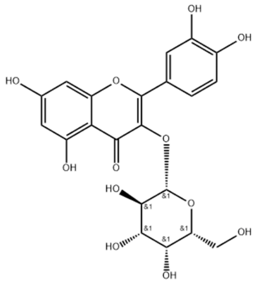
Epimedin A	C39H50O20	861.27882	109059303.725343	110623-72-8	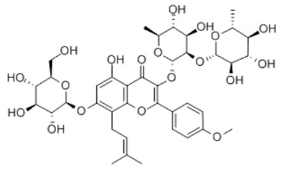
Epmedin C	C39H50O19	845.28477	155605826.859729	110642-44-9	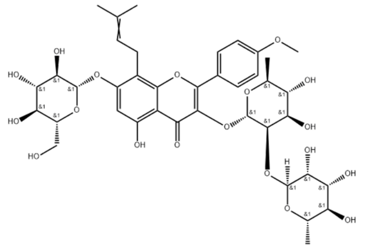
Icariin	C33H40O15	699.22642	197099026.233058	489-32-7	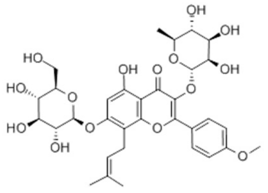
Icariside I	C27H30O11	531.18691	28399616603.7154	56725-99-6	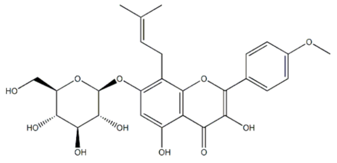
Noricaritin	C20H20O7	355.1185	384947634.626608	5240-95-9	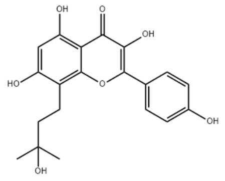
Epimedin B	C38H48O19	831.26858	60627285.6040469	110623-73-9	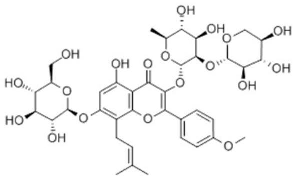
Isoscoparin	C22H22O11	445.11003	6230820.46875	20013-23-4	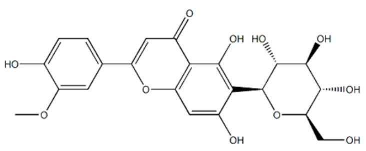

Tricin	C17H14O7	331.08194	2567766894.83364	520-32-1	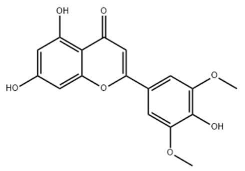
